# A New Method to Reconstruct Recombination Events at a Genomic Scale

**DOI:** 10.1371/journal.pcbi.1001010

**Published:** 2010-11-24

**Authors:** Marta Melé, Asif Javed, Marc Pybus, Francesc Calafell, Laxmi Parida, Jaume Bertranpetit, The Genographic Consortium

**Affiliations:** 1 IBE, Institute of Evolutionary Biology (UPF-CSIC), CEXS-UPF-PRBB, Barcelona, Catalonia, Spain; 2 Computational Biology Center, IBM T J Watson Research, Yorktown, New York, United States of America; 3 CIBER Epidemiología y Salud Pública (CIBERESP), Barcelona, Catalonia, Spain; University of Oxford, United Kingdom

## Abstract

Recombination is one of the main forces shaping genome diversity, but the information it generates is often overlooked. A recombination event creates a junction between two parental sequences that may be transmitted to the subsequent generations. Just like mutations, these junctions carry evidence of the shared past of the sequences. We present the IRiS algorithm, which detects past recombination events from extant sequences and specifies the place of each recombination and which are the recombinants sequences. We have validated and calibrated IRiS for the human genome using coalescent simulations replicating standard human demographic history and a variable recombination rate model, and we have fine-tuned IRiS parameters to simultaneously optimize for false discovery rate, sensitivity, and accuracy in placing the recombination events in the sequence. Newer recombinations overwrite traces of past ones and our results indicate more recent recombinations are detected by IRiS with greater sensitivity. IRiS analysis of the MS32 region, previously studied using sperm typing, showed good concordance with estimated recombination rates. We also applied IRiS to haplotypes for 18 X-chromosome regions in HapMap Phase 3 populations. Recombination events detected for each individual were recoded as binary allelic states and combined into recotypes. Principal component analysis and multidimensional scaling based on recotypes reproduced the relationships between the eleven HapMap Phase III populations that can be expected from known human population history, thus further validating IRiS. We believe that our new method will contribute to the study of the distribution of recombination events across the genomes and, for the first time, it will allow the use of recombination as genetic marker to study human genetic variation.

## Author Summary

Recombination brings together DNA sequences that can be very distantly related, and, thus, quite different from each other. This is often cited as a main hurdle for using recombining regions (that is, most of the genome) to reconstruct sequence phylogeny. We have turned this argument around: chromosomes carrying a similar change in sequence pattern are likely to be descendants of the same recombination event, and thus, related. We have devised an algorithm that detects such changes in sequence patterns and identifies the descendants of a recombination event. After some fine-tuning, we have applied it to sequence data in several human populations and have found that recombination events recapitulate the history of these populations. This opens the possibility of adding recombination to the current allele-based analysis of population structure and history. Our method also provides a tool for the genomic analysis of recombination, both because it pinpoints recombination events rather than just estimating recombination rates, and because, being biased towards more recent events, it can offer a glimpse of the fast evolution of recombination.

## Introduction

Recombination has been lately the focus of much attention. Specifically, much effort has concentrated in trying to understand the extensive variation of the recombination process seen in humans and to unravel the basic mechanisms underlying this variation [1]–[4]. Understanding recombination is an essential step in the path to understanding the structure of the genome, and the strategies needed for searching specific genome regions related to complex traits and diseases. These strategies mostly depend on exploiting linkage disequilibrium; that is, the correlation between markers along the sequence.

In the present study, we have developed a method that allows studying recombination from a new perspective: using the presence or absence of the trace of a particular recombination event in a specific sequence as a genetic marker. Actually, this idea was first proposed by Sir Ronald A. Fisher [5] more than fifty years ago. He introduced the concept of junction [6], and stressed that the breakpoint created by recombination while putting together sequences with different phylogenetic histories carries a signal of a shared history for the descendant sequences. Once created, a junction will be inherited just like a point mutation, and thus can be used as a genetic marker. Moreover, in the same way that several nucleotide states in a chromosome segment configure a haplotype, it is possible to define that several junctions (i.e., the presence of any set of possible recombinations) constitute a *recotype.*

Many different methods have been developed to detect recombination; they are implemented in a number of computer programs listed at http://www.bioinf.manchester.ac.uk/recombination/ and reviewed by [7]. Most of the available methods that tackle presence or absence of recombination, however, are either aimed at placing possible breakpoints or at detecting single recombinant sequences, rather than at an exhaustive search for past recombination events. Moreover, most of them are rather computer-intensive and cannot perform the analysis of a large number of sequences and SNPs. On the other hand, methods implemented in programs such as PHASE [8], [9] and LDhat [10], [11] infer population recombination rates; those, however, do not detect specific recombinations or specify which are the sequences carrying the information of the recombination events.

We have developed a method that is specifically aimed at detecting past recombination events from a set of extant human haplotypes and that is able to tell which are the sequences carrying the information of these events. The method called IRiS (Identifying Recombination in Sequences) performs an extensive screening for recombination within a large amount of markers and sequences in short computing time. IRiS is based on a combinatoric algorithm [12]; details on the efficacy can be found in Parida et. al. [13]. Roughly speaking, the method uses the patterns created by the polymorphic positions in the extant DNA sequences to infer recombinant sequences and to locate the breakpoint. For each run of the algorithm, the output is a set of pattern-based networks, each of which represents a portion of the region analyzed. In those networks, recombination events are represented as nodes having two parental nodes, and the descendant sequences of the recombinant nodes are the recombinants. The method is based on aggregating several runs of the algorithm using multiple sliding windows of different sizes. Adding up the information on the successive runs, we obtain, for each recombination event, a distribution of detections in specific sequences along the SNPs. The highest point of each distribution is the estimated breakpoint location and the sequences carrying the information of that event are the recombinants. Each initial sequence will have then signals of a set of past recombinations (junctions) and the string representing the presence or absence of all possible junctions are the *recotypes*. The final output then will be a set of *recotypes*, one for each initial sequence, and a set of estimated breakpoint locations, one for each recombination event inferred; in a single position, more than one recombination may be retrieved depending on the identity of the parental sequences.

In this paper, we calibrate and validate our method using extensive simulations, which will both give us a proxy for the efficiency of our method and also help us to understand which recombinations are preferentially detected. The simulations used incorporate a model that mimics human demography and variable recombination rates including the presence of hotspots, allowing us to evaluate the performance of IRiS within these regions. Moreover, we compare its performance to known cases of recombination observed by sperm typing or estimated by linkage disequilibrium based methods. Finally, we apply IRiS to reconstruct the recombination history of several gene-free regions on the X chromosome from the HapMap3 dataset [14] to analyze the relationships among those populations using for the first time recombination as a genetic marker.

The applications of the method can be extended to other fields such as basic genetics, recombination dynamics, and the analysis of structure of the genome. IRiS provides in fact a novel tool to understand the past of recombinant genomes.

## Results

### Description of the method

**Figure 1:**
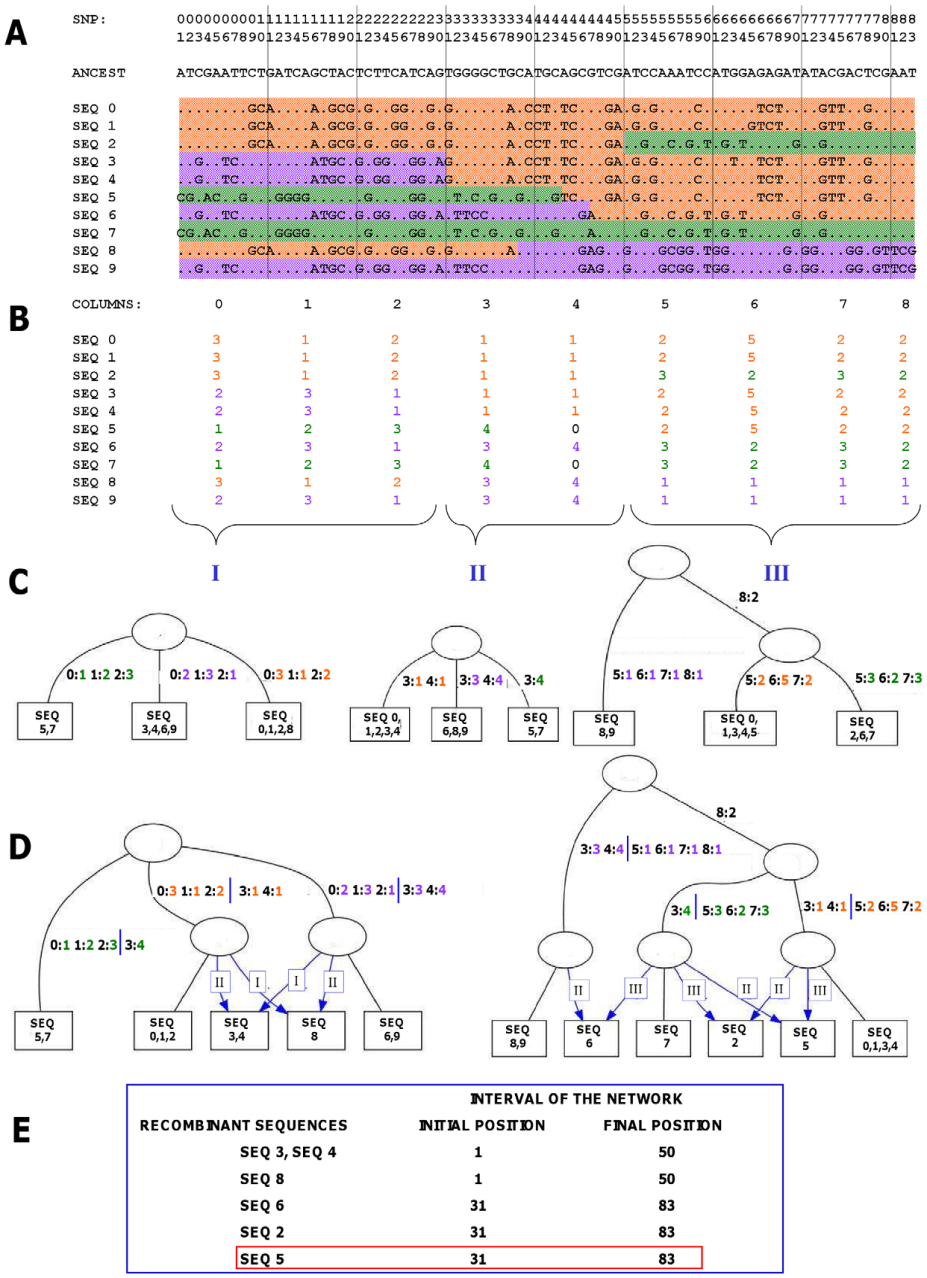
Scheme of the recombination detection process for one run of the algorithm. (A) Input dataset of 10 sequences and 83 SNPs. Colors on sequences represent similar patterns of SNPs, and a change of color along a sequence represents the signal of past recombination events. (B) Recoded matrix. The patterns of SNPs within a column of grain size *n* (10 SNPs in this example) have been recoded into numbers. Those sequences having the same pattern within a column will be assigned the same number. Between columns, numbers represent completely different patterns. Unique patterns are assigned the number zero and will not be considered. (C) Trees one, two and three, constructed based on the recoded matrix. Going from left to right, the recoded matrix is segmented into sets of compatible [30] columns of patterns. Compatibility of columns is checked using a variant of the four gamete test [31] for multi-allelic markers. Each segment is represented as a tree in which the leaf nodes contain the sequences analyzed and the edges contain the patterns inherited, similar to point mutations. Recurrence is not allowed. (D) Networks 1–2 and 2–3 constructed from consecutive trees one, two and three merged pairwise. All the information contained in the two original trees will be present in the compatible network. Recombinant sequences are leaf nodes descending from nodes having two parents, which means that have inherited patterns from two different nodes (similar to an Ancestral Recombination Graph). (E) Information saved for each detected recombination event: the recombinants sequences and the starting and ending position of the network. For a more detailed description of the algorithm see [12]. In red, the recombination event that will be further studied in Figure 2.

IRiS is based on the algorithm described in [12]. Basically, it uses patterns of SNPs of size *n* (grain size) in order to construct a set of consecutive pattern-based networks along the sequence (Figure 1). First, the patterns are recoded into numbers (Figure 1A and 1B), then a set of consecutive pattern-based trees are constructed (Figure 1C) and finally, the information of consecutive trees is merged to construct pattern-based networks (Figure 1D). In those networks, recombination events are represented by nodes having two (rather than one) parental nodes and all subsequent descendants of such biparental nodes are sequences that carry the signal of that recombination event. For each network and for each detected recombination event, the information about which sequences are the recombinants and the starting and ending position of the network is saved (Figure 1E).

**Figure 2:**
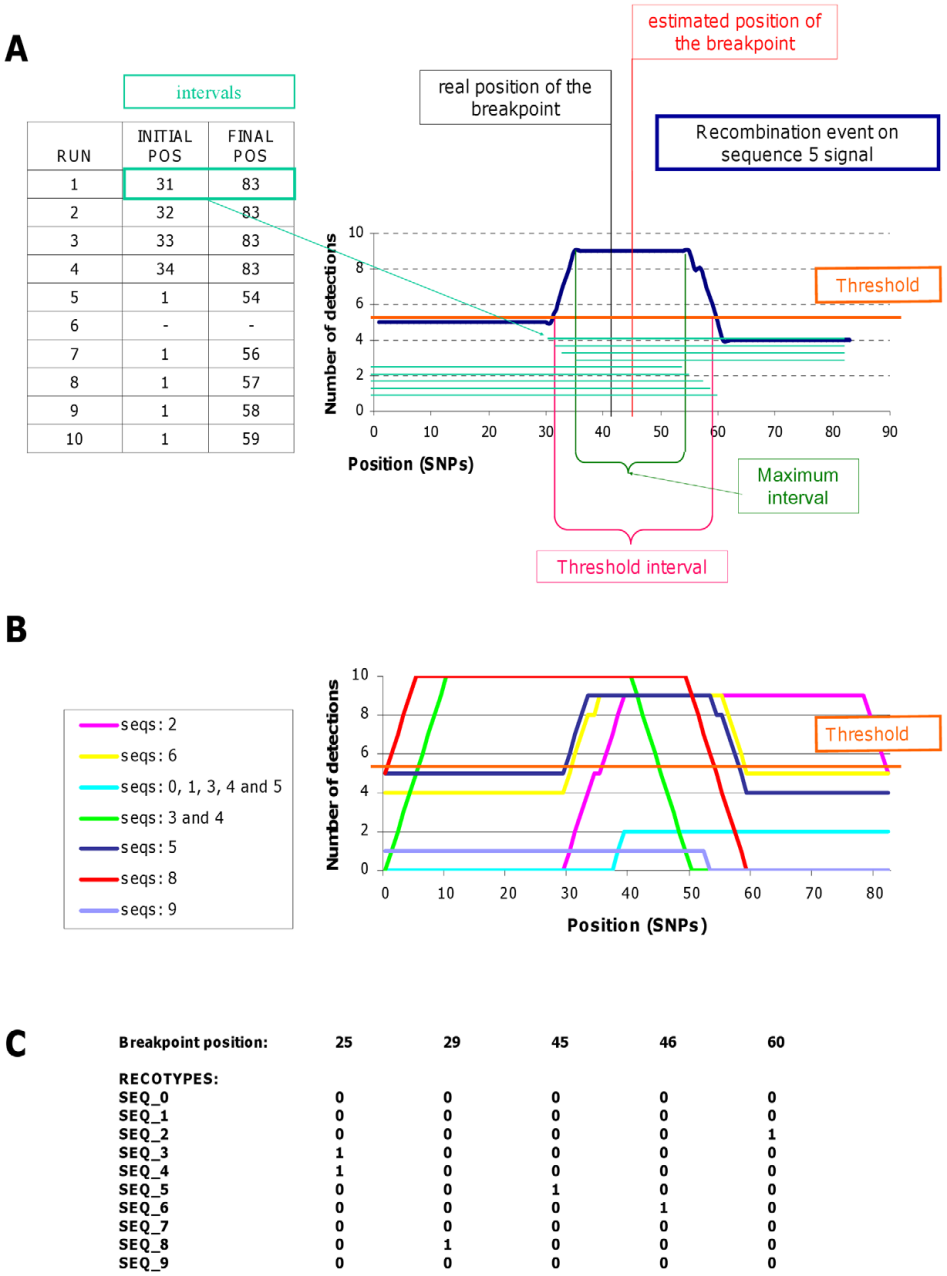
Scheme of the recombination detection process integrating 10 runs of the algorithm. The analyzed dataset is the one shown in Figure 1. (A) Integration of the information of 10 runs regarding the recombination event of sequence 5. For each run of the algorithm, the starting and ending position of the network in which the recombination is detected, is saved. For each run, the size of the first column varies, being 10, 1, 2, 3… up to 9 and therefore the number of runs corresponds to the grain size. At the end, for each recombination event, we have a set of intervals in which it was detected which can be represented graphically as a distribution. The maximum interval represents the region in which the recombination has been seen the maximum number of times. The mean point of the maximum interval is defined as the estimated breakpoint position. The threshold indicates the number of times a recombination has to be detected to be considered as true. The intersection between the threshold and the detection distribution defines the threshold interval in which the algorithm guarantees that the recombination event is located. (B) Integration of the information of all detections for the 10 runs of the algorithm. Each line represents a set of sequences in which the same recombination event has been detected; the distribution of the line shows the number of times the event has been detected along the sequence. (C) Final output of the algorithm: breakpoint positon in the first row, the recotypes in rows and the recombination events detected in columns. The presence of a particular recombination event in a particular sequence is represented as a 1, and absence as a 0. Note that the recotypes represent exactly the coloring of the sequences in Figure 1 and that only recombinations that had a distribution above the threshold are represented in the recotypes.

Since this algorithm divides the haplotypes into SNP patterns of size *n* and the capacity to detect recombination is higher close to the boundaries of a pattern, a sliding window approach is used. Therefore, the algorithm has to be run *n* times and the size of the first column will vary from 1 to *n* across runs. A list of the recombinant sequences and the intervals in which they have been detected is saved. Note that particular recombination events will potentially be detected in different runs of the algorithm and so the same set of recombinants would have different overlapping intervals in which these recombinations have been detected. Adding up the information on the successive intervals, we obtain a distribution representing the number of times a specific recombination has been detected along the sequence (Figure 2A and B). The distribution given by the multiple runs of the sliding window not only helps to narrow the location of the breakpoint, but also defines the certainty of the detection. This allows setting up a threshold on the number of times a particular event had to be detected to be considered a true recombination. The interval of the distribution above the threshold was defined as the threshold interval and the highest point of the distribution is where the breakpoint position is inferred. In case the highest point was a plateau, the inferred breakpoint position would be located in the middle (Figure 2A). The final output of the algorithm is a set of strings, one for each initial sequence, in which the presence or absence of particular recombination events are represented as ones and zeros: those strings are called the recotypes (Figure 2C).

**Figure 3:**
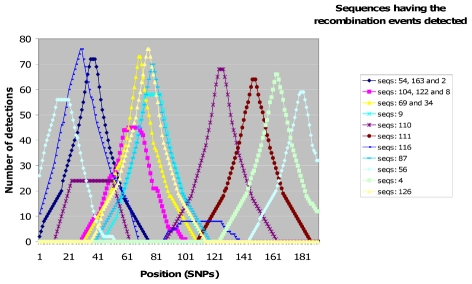
Distribution of the number of detections using the optimal method. Each line represents the distribution of detections for particular recombination events. The dataset corresponds to one COSI simulation. Only those recombinations reaching the threshold will be considered as true events. The pick of each distribution will locate the breakpoint position for each particular recombination event along the sequence. The optimal method (grains 20, 10 and 5 forward and reverse and a threshold of 42) creates narrower maximal intervals in the detection distributions than when only using grain 10.

Finally, using the same approach, we can potentially aggregate detections of multiple runs performed with different grain sizes and also we can run the algorithm in both forward and reverse directions. This aggregation improved significantly the performance of the method (see next section) by increasing the sensitivity and reducing the false discovery rate. Moreover, it allowed a much more precise inference of the breakpoint position since maximum intervals become much narrower (Figure 3).

### Calibration of the method

We sought the best parameter set for IRiS in human sequences by calibrating the program using sequences produced by the coalescent simulator COSI [15]. COSI implements a demographic scenario and a variable recombination rate model that generates sequences that show linkage disequilibrium patterns similar to those found in different human populations (African, African American, European and Asian). Since the location of each simulated recombination event was known, we could measure IRiS performance in terms of false discovery rate, sensitivity, and accuracy in placing the recombination event. Since we wanted to simulate the type of data that are normally available in human databases, we first ascertained the tag SNPs produced by COSI and then applied IRiS.

For each parameter set false discovery rate, sensitivity and accuracy of placement (defined as the empirical 90th percentile of the distribution of the distances between actual and inferred recombination location, in terms of number of SNPs) were averaged over 100 COSI simulations. We first evaluated the impact of varying grain size (5, 10, 15, 20, and 30) and threshold defined as the percentage of algorithm runs in which a particular recombination event has to be detected to be considered as true. Next, we assessed the improvement in results gained by running the algorithm in both forward and reverse directions (Figure S1).

Results showed that both grain size and the threshold affect the false discovery rate. It decreases with increasing grain size, and also varies with threshold, reaching the lowest at thresholds of 60% (Figure S1A). Sensitivity results were basically dominated by threshold: increasing the threshold decreased sensitivity. Intermediate grain sizes (10, 15 and 20) performed better in detecting recombination (Figure S1B). Accuracy of placement was dominated by grain size; the higher the grain size the lower the accuracy. Moreover, by running the algorithm in both forward and reverse directions the accuracy of placement was improved (Figure S1C).

**Figure 4:**
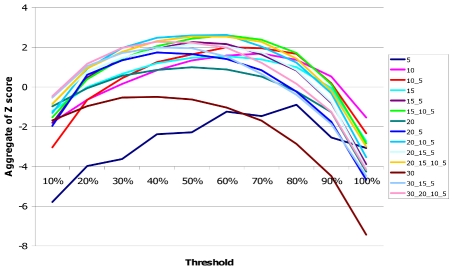
Values of the aggregate Z scores for different settings. Z scores were calculated over mean values for 100 simulations of false discovery rate, sensitivity and 90th percentile of the distance between the inferred breakpoint to the true position. Different colored lines represent different methods, the numbers on the legend inform on the grain size used and whether they combine more than one grain size. All methods are run using a sliding window and forward and reverse. Different thresholds are represented along the X axes. Threshold is defined as number of detections to be considered as true divided by the number of runs of the algorithm.

Since grain size provides a tradeoff between false discovery rate and accuracy of placement, we evaluated combinations of different grain sizes in order to obtain a method that combined an acceptable false discovery rate with high accuracy. For all the methods, we ran the algorithm in both forward and reverse directions (Figure S2). In order to quantify the performance of different methods, we calculated the z-score of the three parameters under evaluation (false discovery rate, sensitivity and accuracy) and added them up to an aggregate z-score. The false discovery rate was given double the weight of sensitivity and accuracy (Figure 4).

The optimal method combined runs with grain sizes 20, 10 and 5 with a threshold of 42. However, other methods had only slightly lower aggregate z-scores. With the optimal method, false discovery rate was 5.79% (below 10% in 90% of the simulations) and sensitivity was 21.15%. The median distance to the breakpoint was 1.59 SNPs: most of the inferred breakpoint locations are not more than 2 SNPs away from their true position.

### Further evaluation of the optimal method

In order to provide a more robust validation and avoid overfitting, we performed 1000 coalescent simulations and estimated false discovery rate, sensitivity and accuracy for the optimal method (Table S1). We also tested the robustness of the optimal method to the SNP ascertainment scheme and SNP density by running several simulations varying the SNP selection process. In all cases, we removed either SNPs with MAF lower than 0.1 or else lower than 0.01 (Table S1); then we either selected SNPs at a certain density as homogeneously spaced along the sequence as possible (all SNPs, 1SNP/1Kb, 1SNPs/2Kb, 1 SNP/5Kb) or we selected tag SNPs with two different methods (pairwise and aggressive; see methods for details). Results are robust to SNP ascertainment and varying SNP density although there is room for improvement especially when SNP density is very high or else, when the SNPs selected include low frequency variants. One of IRiS parameters called mergepats is designed to make the program more robust to events such as recurrent mutation or genotyping errors. Evaluation of the method when the parameter mergepats is active was also performed and results show that the performance of IRiS does not suffer when mergepats is activated (Table S1).

Since each recombination event is defined by a set of descendant sequences and an interval, there is no straightforward definition of true negatives and that is why only false discovery rate and sensitivity were evaluated. In order to estimate the performance of the method in the absence of recombination, we performed 100 blank COSI simulations, with no recombination. The mean number of recombinations detected was less than one per simulation (0.84) and the median turned out to be zero.

Finally we assessed whether some matches between COSI recombinations and IRiS calls were chance events by scoring 100 IRiS outputs against 100 random COSI outputs (Figure S3). The median false discovery rate was 85% and the sensitivity 4%. Accuracy decreased greatly, as the median distance to the breakpoint was 6 SNPs (up from less than 2 SNPs).

### Age of the recombination events detected

We assessed which was the distribution of the age of the recombination events detected by IRiS to estimate the time-frame of the events that our method was able to detect. From all recombinations detected in 500 different simulations, 90% of them occurred between present and 3,205 generations ago. Moreover, the median age of the recombination events detected per simulation was 663 generations (around 13,000 years).

**Figure 5:**
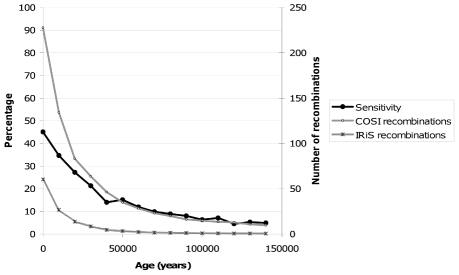
Sensitivity of the optimal method to detect recombinations depending on age. Results plotted are the averaged between 100 simulations. The black curve depicts how sensitivity of IRiS varies with the age of the recombination events (in bins of 500 generations) and follows the left axis. The two gray curves represent the number of recombination events generated by COSI and detected by IRiS and follow the right axis.

We also studied the effect of the age of the recombination events on false discovery rate and sensitivity. When looking across parameter sets, the methods with lowest false discovery rates tended to detect most recent recombination events as average (Figure S4). It is important to highlight that evidence of older recombinations is overwritten by newer ones and hence they leave their trace in relatively shorter segments requiring smaller grain size to be detected which, at the same time, tend to have a higher false discovery rate (Figure S1D). We also calculated the sensitivity of IRiS along time in bins of 500 generations for 100 COSI simulations (Figure 5) using the optimal method. Results show that sensitivity increases with time from past to present going up to 45% for recombination events having occurred in the past 500 generations.

### Influence of the number of recombination events generated by COSI

The number of recombination events affects the performance of IRiS, since we found that false discovery rate correlated with the number of recombinations (r =  0.412; p<10^−6^). A much higher linear correlation was found for sensitivity (r = 0.734; p<10^−6^), which was even higher if fitted to a logarithmic curve (r = 0.925; p<10^−6^), meaning that IRiS reached a plateau beyond which even if the number of recombinations generated increased, IRiS did not detect them. Interestingly, when comparing the number of recombinations detected by IRiS across the simulated datasets with the mean recombination rates estimated by LDhat [16], they were found to be significantly and linearly correlated (0.968; p<10^−6^). This suggests that such a high amount of recombinations does not leave traces on the patterns of linkage disequilibrium and then it is neither detectable by LDhat nor IRiS. The saturation is only achieved (both in IRiS and LDhat) with a very large number of recombinations, in the order of one and half orders of magnitude higher than the average recombination rate of the genome (data not shown); this value may only be achieved by a very strong hotspot. Finally, the distance between the actual and IRiS inferred breakpoint was also correlated with the number of recombinations (r =  0.352; p<10^−6^), meaning that the accuracy in the placement of recombinations decreases when there are large number of recombinations.

### Correlation with inferred recombination rates by sperm typing and LDhat

**Figure 6:**
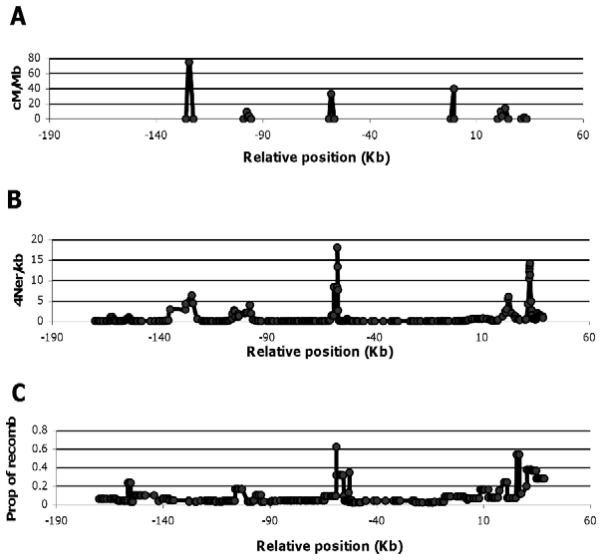
Recombination rates inferred from sperm typing, LD-based methods and IRiS on the MS32 region. (A) Inferred recombination rates based on sperm typing information; figure adapted from the figures in [17] in which they calculate recombination rates through sperm typing. (B) Recombination rate inferred by LDhat. (C) Number of recombination events detected by IRiS using the optimal method. Recombination rates inferred in (A) are based on a single individual whereas recombination rates inferred at (B) and (C) are based on the same population data. Position zero marks the location of the minisatellite MS32.

We compared IRiS performance against linkage disequilibrium based estimates of recombination rates in a region where direct sperm-typing rates were also available. A region of chromosome 1 near the MS32 minisatellite contains some recombination hotspots that were both observed through sperm typing and inferred with different statistical methods [17]. The population of European origin (CEU) from HapMap Phase 2 was used as a surrogate for British samples studied by [17] and population recombination rate was inferred using LDhat [16]. The same data was analyzed by IRiS with the optimal method. The number of recombinations detected by IRiS closely matched both the recombination hotspots detected by sperm typing and specially the estimated recombination rate between each pair of SNPs inferred by LDhat (Spearman correlation coefficient r = 0.604; p<10^−6^ for the estimated recombination rate by LDhat) (Figure 6). Nonetheless, it is interesting to note clear discrepancies between sperm typing analysis and both recombination rate estimated and specific recombinants detected by IRiS, a fact initially discussed in Jeffreys et al (2005) [17].

#### Recombining real data *in silico*

**Figure 7:**
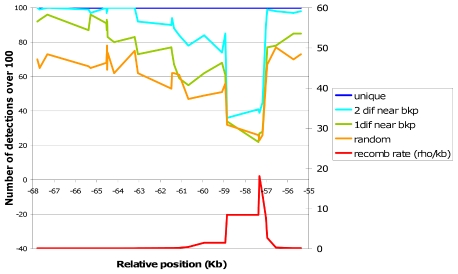
Sensitivity of the optimal method evaluated *in silico*. The plot shows the number of times *in silico* recombination events along the sequence were detected by IRiS depending on the breakpoint location. Different colors indicate different ways to produce the recombinant sequence, from light gray to black: “random” indicates that parental haplotypes were taken at random, “1dif near bkp” indicates that parental sequences had to be different near the breakpoint region (plus minus 10 SNPs), “ 2 dif near bkp” indicates that parental sequences had to be different near the breakpoint regions at both sides of the breakpoint, and “ unique” indicates that the parental sequences had to be different near the breakpoint region and the recombinant sequence had to be unique within the breakpoint region. Below, the recombination rate estimated by LDhat is shown, following the right axis.

A study on the capacity of the optimal method to detect recent recombination events was performed through *in silico* recombination simulations with real sequences (same dataset as in the previous section). This allowed us to assess the characteristics that a particular recombination event should have in order to be detected by IRiS, and also to evaluate IRiS when working with real data. We performed the same analysis several times varying the process of selection of the two parental haplotypes (Figure 7).

Results showed that IRiS performs much better in detecting recombination events that occur between parental sequences that differ in a certain number of SNPs and have those differences close to the breakpoint location. If the recombinant sequence is unique, then there is less room for confusion and IRiS performs even better. Finally, we see that within the hotspot region, even if the recombinant sequence is unique and the two parental sequences are different, the sensitivity is not optimal. That could be due to the fact that since nearly all sequences are recombinant, IRiS detected different recombination events across runs, and many of them did not reach the necessary threshold to be counted as true. Actually, if all events are considered regardless of the number of times they have been detected, the sensitivity increases to 100% (Figure S5).

#### Gene conversion, recurrent mutation and genotyping errors

Gene conversion, recurrent mutation and genotyping errors were not modelled in the COSI simulations. We used *in silico* simulations to evaluate how these factors could affect IRiS performance. We evaluated the parameter mergepats which if activated, patterns of SNPs that differ in one SNP position are considered as the same (see methods for details).

We performed 12,000 *in silico* simulations, 1000 for each scenario, to simulate gene conversion involving different number of SNPs and recurrent mutation (which will behave as a putative genotyping error as well). Using the same dataset as in the previous section (see methods for details), we evaluated how many times each of these events was detected as either one or two recombination events or not detected at all (Table S2).

Results show that, first, gene conversion will not have an impact on IRiS results since the majority of gene conversion events involve a very small number of SNPs [4], and under this scenario, they are ignored by IRiS. Second, we have seen that some recurrent mutation events can be detected as recombinations. Although we know that recurrence does not occur so frequently in the nuclear DNA, it may have some impact on IRiS performance in regions with high mutation rate. It is clear that activating the mergepats parameter in all such cases will help to create more reliable analysis.

#### Dealing with phasing errors

Since IRiS uses haplotype data, we evaluated its robustness to phasing errors using similar *in silico* simulations as in the previous section. A phasing error is simulated as two reciprocal recombination events occurring in the same position in the two homolog chromosomes of an individual. Results show that most of phasing errors will not affect IRiS performance, either because they are ignored (57.3%) or else because they can be detected as reciprocal recombinations (30.4%) and be discarded from the output.

In order to study the impact of phasing errors further, we selected 18 regions from the X chromosome from HapMap phase 3 project (see methods) and compared the number of recombinations detected by IRiS using 537 male X chromosomes (in which the phase is known) with 537 female X chromosomes. The female chromosomes were phased independent of the male chromosomes using two different softwares: PHASE [18], [19] and fastPHASE [20]. We also performed a post-processing of the output by removing pairs of recombination events occurring in the two homologous chromosomes of a female in the same position (Table S3).

Results show that when sequences are phased using PHASE, no differences in the number of recombinations can be found between males and females (Wilcoxon test; p =  0.992 ); even when grouping the number of recombinations detected in bins of 5 SNPs along the sequence (Wilcoxon test; p =  0.795). Conversely, when phasing with fastPHASE, the number of recombinations detected in the female sample is significantly higher in females both when calculating it per region (Wilocxon test; p<10^−6^) and in bins of 5 SNPs (Wilcoxon test; p = 0.000292).

Regarding the post-processing in which recombinations occurring in the two homolog chromosomes of the same individual are removed, there is a difference between the three datasets. In the male dataset (in which all recombinations that may be removed are not phasing errors), 5.59% of the recombinations are removed. In the female dataset phased with PHASE, 3.37% of the sequences are removed, whereas for the fastPHASE phased dataset 9.70% of the sequences are removed. This may be indicating that some of the sequences that are removed in the fastPHASE dataset are indeed phasing errors.

#### Using recombinations as genetic marker in human population genetics

**Figure 8:**
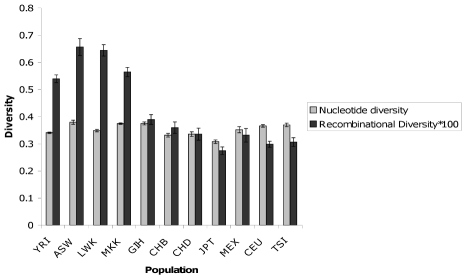
Nucleotide and recombination diversity. Values were calculated for each of the populations based both on haplotypes and recotypes for the 18 regions. Values of recombination diversity have been multiplied by 100 to make them comparable.

A search for human X-chromosome regions harboring more than 80 SNPs and not containing known genes, copy number variants or segmental duplications (see Methods) yielded the 18 regions shown in Table S4. Overall, they span slightly more than 7 Mb, and contain 2054 SNPs that were genotyped in 537 male X chromosomes of the HapMap [14] Phase 3 project. We selected the X chromosome in males in order to avoid phasing errors that would mimic recent recombination events. We run IRiS over the 18 regions independently using the optimal method; we inferred a total of 3598 recombinations. Thus, after running IRiS in a set of haplotypes, we obtained a set of *recotypes*, each of them representing the recombination history of each initial chromosome with the putative position of each of the recombination events. We calculated the nucleotide diversity over the 18 regions together, and we also calculated the recombination diversity by doing the same process with the *recotype* data (Figure 8). Because of the ascertainment bias in SNP selection in Hap Map 3, nucleotide diversity values did not show any specific pattern at the continental level; but recombination diversity did, having a much higher diversity within African populations. This suggests that the recombinational diversity measure is not affected by the SNP ascertainment bias.

**Figure 9:**
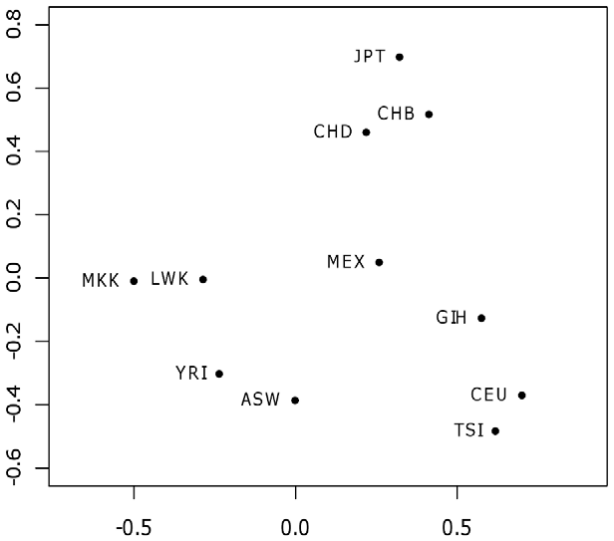
First and second components of the Principal Components Analysis. Only recombinations present in at least in two individuals were taken for the analysis. The first component explained 18.03% of the variance and the second component 14.53%.

We next analyzed the geographical structure of recombination events by means of two different statistical analyses: Principal Component Analysis (PCA) and Multidimensional Scaling (MDS). Results of the PCA analysis for component 1 and 2 can be seen in Figure 9. The first component separates African from non–African populations, with the African-Americans in an intermediate position. The second component separates European from East Asian populations leaving the Mexican in between them and the South Asians closer to the Europeans. Interestingly, the second component also separates Western Africans (Yoruba) and African-Americans, from the Eastern Africans (Maasai and Luhya).

MDS was based on a recombination distance matrix among the eleven populations and showed very similar results as for the PCA (Figure S6) where the first dimension would separate between African and non-African populations and the second one would separate between European and East Asian. The stress in the MDS was significantly lower than expected for a random collection of points [21]. Finally, beyond the graphic representation of population differentiation, we calculated the correlation between the genetic distances based on SNP allele frequencies among the eleven populations with the recombination distance matrix and the mean correlation coefficient was 0.756 (p-value<10^−6^).

The geographical structure found by these analyses is consistent with that produced with allele frequencies in classical markers, short tandem repeats [22], single nucleotide polymorphisms [23], [24], [25] and haplotypes or CNV [25] to the point that it can be considered a general consensus.

## Discussion

We present here a novel method that is able to extensively detect and place historical recombination events. We have calibrated and validated it specifically for human variation through simulations, but it could be easily adapted to other species provided that basic models for demography and recombination exist. An optimal parameter set has been defined based on three performance criteria: false discovery rate, sensitivity, and accuracy in placing the breakpoints. Given that different parameters had contrasting effects (a decrease in false discovery rate is often accompanied by a decrease in sensitivity, for instance), a compromise was sought and the best parameter set was the one that combined grain sizes of 20, 10 and 5 run forward and reverse with a threshold of 42. It should be noted, though, that some applications may require a different balance; for instance, greater accuracy may be needed, and the parameter set can be adapted to accommodate these different requirements.

Recombination rate varies across the genome [9], [26] and the number of actual recombinations in the sample history will affect IRiS performance. IRiS tends to saturate with a growing number of actual recombinations per sequence, but its behavior mimics that of linkage disequilibrium based recombination rate estimation algorithms such as LDhat [11]. It is possible then that a large number of recombinations will not generate a corresponding increase in the information that can be extracted either from patterns or from linkage disequilibrium. This imposes a limit on IRiS, and may be particularly restrictive in recombination hotspots, where repeated recombinations in the same location will erase the signal of older events, letting IRiS to recover a lower number of events than the ones that really occurred. On the other hand, we should also take into account the possible confounding role of homoplasy. For two different recombination events to be counted as one they not only have to share the breakpoint location but they should have similar SNP patterns flanking the breakpoint. The fact that the false discovery rate is low indicates that there are not many cases in which IRiS considers two sequences as descendants of the same recombination event and they are not.

We have seen that sensitivity is higher for more recent recombination events both in those events generated in the last 500 generations (∼10,000 years) in coalescent simulations, or in those generated instantly *in silico* from extant human sequences. On the contrary, older recombinations are harder to detect. Two factors may account for this bias: older recombinations may have been partially erased by subsequent recombination events, and recombinations involving more divergent parental sequences are easier to detect; those more ancient may happen among identical sequences without leaving any footprint. While this bias implies that recombination events happening deeper in human history may be difficult to recover, it also provides a tool that connects populations that diverged recently, or may even signal relatively shallow genealogical links between individuals, and could be used in the emerging field of genetic genealogy.

Several factors such as gene conversion, recurrent mutation, genotyping errors, phasing errors, or SNP ascertainment may affect the performance of IRiS and in this study we have extensively evaluated the impact of them all. First, we have shown that gene conversion has higher impact as more SNPs are involved in the process. Since gene conversion typically involves very small regions [4], the majority of gene conversion events will involve one SNP and consequently will be ignored by IRiS. Secondly, we have seen only very few recurrent mutation events or genotyping errors being falsely detected as recombinations, especially if the mergepats parameter is activated. Recurrent mutations do not occur so frequently in the nuclear DNA, but there may be regions in which mutation rate is higher than average and patterns similar to those generated by recombination may appear. In those cases the mergepats function must be activated. Third, we have shown that according to *in silico* simulations, most of phasing errors will not affect IRiS performance, either because they are ignored (57%) or else because they can be detected as reciprocal recombinations (30%) and be discarded of the output. It is important to highlight, however, that if the phasing is not accurate there may be some fake recombinations in the dataset. If this is known a priori, we suggest the option of post-processing the detected recombination and remove those that look like phasing errors. In any case, special care should be taken to decide which software is used when inferring the phase of haplotypes: we believe that datasets with high phasing quality are needed in order to run IRiS on them. Finally we have shown that IRiS is able to perform well under very different SNP density scenarios and SNP ascertainment processes. It is important to note however that the method is not optimized to analyze resequencing data and incorporate information on rare variants. Further studies on the optimal parameters to use in this end should be performed. Conversely, IRiS would not be able to deal with completely uncorrelated SNPs since it is based on the patterns of SNPs created due to LD to identify recombination events.

When applying our method to population genetics, using a set of regions in the X chromosome in HapMap Phase III data, we found that the ascertainment of the SNPs present in HapMap resulted in similar nucleotide diversities in all populations, whereas the recombination diversity values were not affected by it. Higher recombination diversity was found in African populations. If nucleotide diversity were different across populations, this could be attributed to a higher capacity of IRiS to detect recombination within more diverse populations. However, since we have seen that the nucleotide diversity in HapMap Phase III is similar across populations, we can conclude that African populations contain indeed more recombination events than the other populations, as expected from their larger long-term effective population size. This is a further validation of the method.

Moreover, the ASW (Afro-Americans) is the population with the highest recombinational diversity. This could be explained by it being an admixed population and because some of the recombination events may be clearer since the two ancestral sequences that recombined may come from very different populations. It has to be stressed that this admixed population has been included in our validation procedure and thus, we have already taken into account the possible effects on IRiS performance due to admixture. The possible use of IRiS to detect and analyze past admixture deserve future attention.

Two different statistical methods that plot the relationship between populations based on their shared recombination events as detected by IRiS have produced results that are strikingly similar to the a priori expectations based on what is known about human population history. This can be taken both as a validation of our method, and as a pilot project for the applicability of IRiS to human population data. We believe that the use of recombination events in conjunction with the standard methods based on SNP and haplotype frequencies [25] will allow extracting the most information from genetic data in the reconstruction of human population history.

In conclusion, we have presented a method that is able to extensively retrieve historical recombination events from a set of extant human haplotypes and point out which are the sequences that contain the information on those events. We also have shown a potential application of the method in population genetics: the use of recombination as a genetic marker that can complement present methods. Finally, we believe that this method will have a whole set of applications beyond population genetics in fields such as the study the recombination dynamics and the recombinational differences between populations, or the study of the mechanisms that have given rise to our recombinant genomes.

## Materials and Methods

### Coalescent simulations pipeline

Simulations were performed using the coalescent simulator named COSI (Shaffner et al. 2005) with the Best-Fitting Model parameters which simulate datasets that closely resemble human data. We took a sequence length of 250,000 bp and a sample size of 50 sequences per population for all populations except the African population, for which the sample size was 60.

In the first phase of the calibration, we evaluated IRiS performance over 100 random simulations having more than 80 SNPs. The number of simulations was then increased to 1000 in order to establish more robust results. For each simulation, the evaluation process was as follows:

First, the COSI best fit model was run; the information of all recombination events created, their descendant sequences, and the age and exact position of the breakpoint was saved.

Second, the SNP ascertainment was performed; SNPs with MAF lower than 10% and non-tagSNPs were removed from the sample. TagSNPs were selected using Haploview with pairwise option (Barrett et al., 2005) and *r^2^* >0.8.

Third, recombinations that took place between identical parental sequences in at least one side of the breakpoint were removed, since they are impossible to detect as recombinants and will be not be considered further (note that those events will not be used to compute sensitivity values).

Fourth, IRiS was run on the dataset created by COSI. Each detected recombination is defined as a set of recombinant sequences and a breakpoint interval defined by IRiS, (maximum of the distribution). Detection is considered correct if there exists a recombination event within the COSI simulation that has exactly the same descendant sequences and it occurred within the interval defined by IRiS. We allow IRiS to detect a subset of the descendants, of a particular recombination event created by COSI, if there has been a younger recombination that masks the trace of the older one in some of the descendant sequences.

Finally, false discovery rate, sensitivity and the 90th percentile for the distance from the inferred to the real location are computed.

All correlations performed on the results of the simulations were calculated by means of the SPSS software (version 15.0).

### Correlation with inferred recombination rates by LDhat and sperm typing

The study of the correlation between IRiS results and hotspots inferred from recombination events detected through sperm typing was performed based on HapMap Phase 2 data (www.hapmap.org). We downloaded SNP genotypes of the HapMap Phase 2 release #21 for the CEU population for the SNPs present in the same region studied by Jeffreys et. al (2005) [17] in chromosome 1 and we obtained a total of 120 chromosomes and 365 SNPs.

Inference of the recombination rates was performed by means of the coalescence-based algorithm implemented in the LDhat package [10], [11] using the program rhomap [16]. The parameters used were the recommended in the user's guide such as 1,100,000 iterations for the rjMCMC procedure, 100,000 iterations for the burn-in. Before using Rhomap, a lookup table file was created from a pre-computed table taken from http://www.stats.ox.ac.uk/~mcvean/LDhat/instructions.html which assumed a theta of 0.001 per site. Recombination rates were calculated for each pair of SNPs as the median of five runs of rhomap. Sperm typing recombination rate estimates were taken directly from the figures in [17].

The IRiS proportion of recombination was calculated based on the threshold interval normalized by the length of the interval in bp. Each recombination contributed equally to all SNPs in the inferred breakpoint interval a value inversely proportional to the length of the interval in bp. To check sensitivity to linkage disequilibrium, raw IRiS detections were added up across all the algorithm runs. Note that in this case each inferred breakpoint interval will be the length of the network in which the recombination was detected.

### *In silico* simulations

In *silico* recombinations were created using the same region on chromosome 1 near the MS32 minisatellite from HapMap Phase 2 used in the previous section. We selected 30 SNPs within the central region that contained one of the defined by sperm typing hotspots and created 100 different recombination events at each of the SNPs locations (3000 simulations overall). Each event was created by taking two parental sequences from the dataset at a time, recombining them, and putting back the recombinant sequence together with the parental sequences. A positive detection was defined when the recombinant sequence was detected as a unique recombinant and the predicted interval contained the true breakpoint location.

We performed the same analysis several times varying the process of selection of the two parental haplotypes. First, they were chosen at random, second, they were only taken if they were different in at least one position within the breakpoint region (defined as a 10 SNP distance of the breakpoint), third, they were taken if they were different in at least one position on each side of the breakpoint within the region, and finally, we would only consider for the analysis those events that created recombinant sequences that was unique within the breakpoint region

*In silico* gene conversion simulations were created by randomly taking two chromosomes, transmitting 1, 3, 5 or 10 SNP variants from one chromosome to the other and adding them back to the original dataset. Recurrent mutations were simulated by choosing a chromosome and a SNP position at random, and changing it to the other allele. Finally, phasing errors were created by taking two chromosomes at random, generating a reciprocal recombination and putting back the two chromosomes in the initial dataset.

We considered that two recombinations to be the putative product of a phasing error if there appeared two recombination events in the two homologs of an individual at a distance of less than 6 SNPs.

The mergepats parameter is implemented in IRiS when defining groups of SNP patterns of size g (which in the optimal method will be 5, 10 and 20). If mergepats is activated, patterns that differ in one SNP will be considered as the same. This is performed hierarchically first by taking the most frequent pattern and merging it with all patterns that are at edit distance one of it. Then the second most frequent pattern will be merged with all patterns being at edit distance of one, and so on. In this way, we avoid merging all patters of a sample into a single one.

### Region selection

The whole X chromosome was screened in order to find the optimal regions for our analysis. Regions at least 50 Kb distant from known genes, copy number variants and segmental duplication, and containing at least 80 SNPs genotyped in the 11 populations from the HapMap Phase 3 release #1 (www.hapmap.org) were sought. These conditions were meant to avoid selection, genotyping errors, and to ensure sufficient precision to detect recombination. A complete list of all the positions of the genes in X chromosome was retrieved from Ensemble 37 using BioMart (http://feb2006.archive.ensembl.org/Homo_sapiens/martview). The coordinates of segmental duplications were retrieved from the Segmental Duplications Database (http://humanparalogy.gs.washington.edu/) and copy number variants and indels from the v5 release of the Database of Genomic Variants (http://projects.tcag.ca/variation/). All positions were based on NCBI Build 35. Equivalent positions from the HapMap Phase 3 SNPs in build 36 were found by querying table *SNP125* from the UCSC database (http://genome.ucsc.edu/cgi-bin/hgTables). Average recombination rate for each of the regions was calculated using the program rhomap [16] in the same way as for the sperm typing region (Table S4).

### X-chromosome genotypes

SNP genotypes for the X chromosome were obtained from the HapMap website (www.hapmap.org). We downloaded SNP data of the HapMap Phase 3 release #1 for the eleven populations: ASW (African ancestry in Southwest USA), CEU (Utah residents with Northern and Western European ancestry from the CEPH collection), CHB (Han Chinese in Beijing, China), CHD (Chinese in Metropolitan Denver, Colorado), GIH (Gujarati Indians in Houston, Texas), JPT (Japanese in Tokyo, Japan), LWK (Luhya in Webuye, Kenya), MEX (Mexican ancestry in Los Angeles, California), MKK (Maasai in Kinyawa, Kenya),TSI (Tuscans in Italy), YRI (Yoruba in Ibadan, Nigeria). Only SNPs genotyped in all populations were used in further analysis.

To avoid phasing errors, only males were selected for recombination analysis; heterozygote positions, which are expected to be erroneous, were considered as missing values (only 0.02% of the positions were heterozygous). Individuals with >5% missing genotypes (22 in total) were discarded; the rest of the missing values were imputed using fastPHASE [20]. Thus, our final panel consisted of 88 MKK, 43 LWK, 88 YRI, 34 ASW, 42 GIH, 40 CHB, 21 CHD, 42 JPT, 25 MEX, 74 CEU, 40 TSI, for a total of 537 X chromosomes.

To obtain the equivalent number of sequences from females we followed the same procedure as males and selected 537 sequences from the same populations in order to match the male dataset. We phased and imputed the missing positions using two different softwares: PHASE [18], [19] and fastPHASE [20].

### Statistical analysis

Nucleotide diversity was calculated using DnaSP [27] having previously merged the sequences of each of the 18 regions respectively. Recombinational diversity was calculated based on recotype information in the same way as nucleotide diversity. PCA and MDS were done using the R package [28]. For Principal Component analysis (PCA), the input matrix consisted on the recombination events present at least in two individuals as variables and the proportion of chromosomes per population carrying each event as cases. As the values were non normalized, the correlation matrix was used to perform the PCA.

For the MDS analysis, the recombinational distance (D_AB_) between populations A and B was computed as:
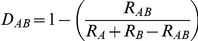



where R_AB_ was the number of recombinations shared between population A and B, R_A_ was the number of recombinations occurring in individuals in population A, and R_B_ the number of recombinations having occurred in individuals of population B. Only those recombinations found in at least two different populations were considered.

*F_ST_* calculations were performed using Arlequin software version 3.1 [29] and so was the Mantel's test used to compare the SNP-based *F_ST_* matrix with the recombinational distance matrix.

## Supporting Information

Figure S1 Mean values taken from the analysis of 100 simulations with different IRiS settings: grain sizes (5, 10, 15, 20 and 30), different thresholds, defined as number of detections to be considered as true divided by the grain size or the double of the grain size in the cases in which the algorithm is run in two directions. For each setting the algorithm could be run only on the forward direction (F) or in both directions (FR). Figure S1A False discovery rate (%). Figure S1B Sensitivity (%). Figure S1C 90% confidence interval of the distance (measured in number of SNPs) between the inferred breakpoint position and the real location. Figure S1D, median age of the detected recombinations.
(0.68 MB DOC)

Figure S2 Mean values taken from the analysis of 100 simulations with different IRiS settings that combine different grain sizes (indicated with different colors), different thresholds (defined as number of detections to be considered as true divided by the sum of the different grain size and multiplied by two since the algorithm is run in the two directions). All settings included running the algorithm in the two possible senses. Figure S2A False discovery rate (%). Figure S2B Sensitivity (%).Figure S2C. 90th percentile distance from the breakpoint location measured in number of SNPs.
(0.53 MB DOC)

Figure S3 Plot showing the relationship between the false discovery rate and the number of COSI simulations under a scenario in which IRiS is given a different dataset than the one used to compare it with the COSI results.
(0.02 MB DOC)

Figure S4 Each dot represents mean values of false discovery rate and median age of the detected recombinations taken from the analysis of 100 simulations with different IRiS settings that combine different grain sizes (indicated with different colors) and different thresholds. All settings included running the algorithm in the two possible senses.
(0.29 MB DOC)

Figure S5 Plot showing values of the number of times in silico recombination events were detected by IRiS run with no threshold depending on the breakpoint location along the sequence. Different colors indicate different ways to produce the recombinant sequence, from light gray to black: “random” indicates that parental haplotypes were taken at random, “1dif near bkp” indicates that parental sequences had to be different near the breakpoint region (plus minus 10 SNPs), “2 dif near bkp” indicates that parental sequences had to be different near the breakpoint regions at both sides of the breakpoint, and “unique” indicates that the parental sequences had to be different near the breakpoint region and the recombinant sequence had to be unique within the breakpoint region. Below, the recombination rate estimated by LDhat is shown, following the right axis.
(0.15 MB DOC)

Figure S6 MDS 2D plot based on a recombinational distance matrix. The stress is 0.081 which is below the 0.16 stress obtained with 1% probability with random data sets (citation: Sturrock K, Rocha J (2000) A Multidimensional Scaling Stress Evaluation Table. Field Methods 12: 49-60).
(0.03 MB DOC)

Table S1 Evaluation of IRiS with the optimal parameters for different SNP ascertainments. SNP selection process is explained in the methods section. Mean SNP density values are calculated over all simulations.
(0.04 MB DOC)

Table S2 Percentage values on the number of times each of the simulated event is either not detected, detected as 1 recombination or as 2 recombinations. The percentage values are calculated over 1,000 in silico simulations.
(0.03 MB DOC)

Table S3 Number of recombinations detected in each of the 18 regions in the male dataset, female dataset and female dataset when removing putative phasing errors. Females were phased using both PHASE and fastPHASE without using male phase information.
(0.05 MB DOC)

Table S4 The main characteristics of 18 X-chromosome regions. From left to right: start position and end position in base pairs (based on NCBI Build 36 assembly), length of each in base pairs, number of SNPs (N SNPs), number of haplotypes (N haplo), recombination rate calculated by means of Ldhat, Number of recombinations detected, number of recotypes, average number of recombinations detected by IRiS per Kb.
(0.06 MB DOC)
