# Supplementary material for: A New Method to Reconstruct Recombination Events at a Genomic Scale
Source: PLoS Comput Biol. 2010 Nov 24;6(11):e1001010. doi: 10.1371/journal.pcbi.1001010 (PMC2991245; doi:10.1371/journal.pcbi.1001010)
Supplement: Figure S1 — Mean values taken from the analysis of 100 simulations with different IRiS settings: grain sizes (5, 10, 15, 20 and 30), different thresholds, defined as number of detections to be considered as true divided by the grain size or the double of the grain size in the cases in which the algorithm is run in two directions. For each setting the algorithm could be run only on the forward direction (F) or in both directions (FR). Figure S1A False discovery rate (%). Figure S1B Sensitivity (%). Figure S1C 90% confidence interval of the distance (measured in number of SNPs) between the inferred breakpoint position and the real location. Figure S1D, median age of the detected recombinations. (0.68 MB DOC) [file pcbi.1001010.s001.doc]

Figure S1A

Figure S1B

Figure S1C

Figure S1D
