# Supplementary material for: A New Method to Reconstruct Recombination Events at a Genomic Scale
Source: PLoS Comput Biol. 2010 Nov 24;6(11):e1001010. doi: 10.1371/journal.pcbi.1001010 (PMC2991245; doi:10.1371/journal.pcbi.1001010)
Supplement: Figure S2 — Mean values taken from the analysis of 100 simulations with different IRiS settings that combine different grain sizes (indicated with different colors), different thresholds (defined as number of detections to be considered as true divided by the sum of the different grain size and multiplied by two since the algorithm is run in the two directions). All settings included running the algorithm in the two possible senses. Figure S2A False discovery rate (%). Figure S2B Sensitivity (%).Figure S2C. 90th percentile distance from the breakpoint location measured in number of SNPs. (0.53 MB DOC) [file pcbi.1001010.s002.doc]

Figure S2A

Figure S2B

Figure S2C
