# Supplementary figures and images for: A New Method to Reconstruct Recombination Events at a Genomic Scale
Source: PLoS Comput Biol. 2010 Nov 24;6(11):e1001010. doi: 10.1371/journal.pcbi.1001010 (PMC2991245; doi:10.1371/journal.pcbi.1001010)

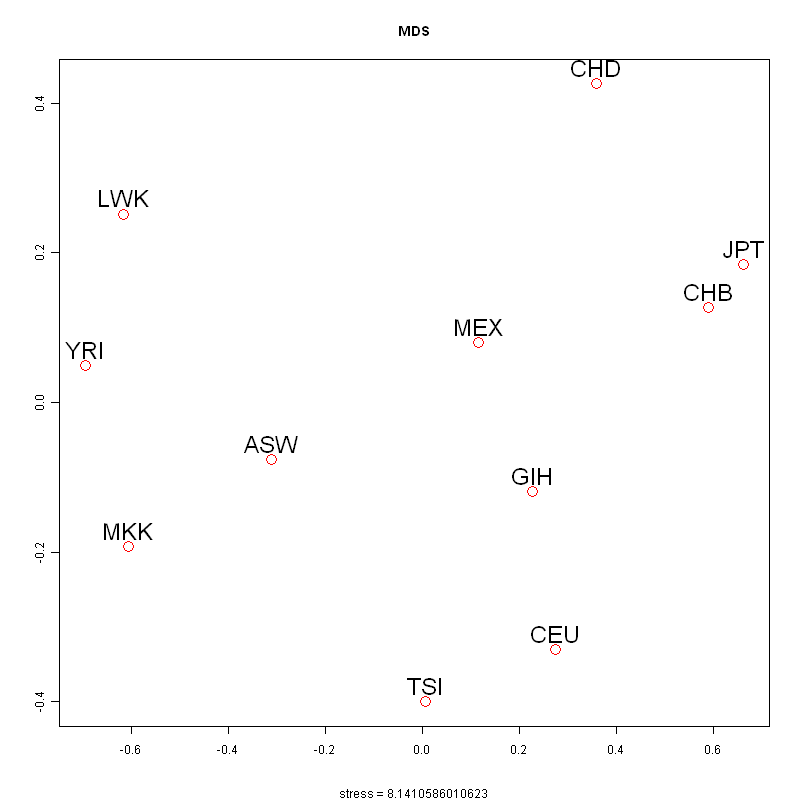

Supplement: Figure S6 — MDS 2D plot based on a recombinational distance matrix. The stress is 0.081 which is below the 0.16 stress obtained with 1% probability with random data sets (citation: Sturrock K, Rocha J (2000) A Multidimensional Scaling Stress Evaluation Table. Field Methods 12: 49-60). (0.03 MB DOC) [file pcbi.1001010.s006.doc]
