# Supplementary material for: A New Method to Reconstruct Recombination Events at a Genomic Scale
Source: PLoS Comput Biol. 2010 Nov 24;6(11):e1001010. doi: 10.1371/journal.pcbi.1001010 (PMC2991245; doi:10.1371/journal.pcbi.1001010)
Supplement: Table S1 — Evaluation of IRiS with the optimal parameters for different SNP ascertainments. SNP selection process is explained in the methods section. Mean SNP density values are calculated over all simulations. (0.04 MB DOC) [file pcbi.1001010.s007.doc]

| SNP selection | mergepat  parameter | minimum MAF | nº of runs | length sequence | mean SNP density | false discovery rate (%) | sensitivity (%) | 90% conf interval |
| --- | --- | --- | --- | --- | --- | --- | --- | --- |
| TAG aggressive | inactive | 0.1 | 69 | 400 Kb | 1/ 2758 bp | 5.64 | 18.61 | 4.75 |
| TAG pairwise | inactive | 0.1 | 69 | 400 Kb | 1 / 2079 bp | 5.52 | 19.18 | 4.80 |
| 1SNP/5Kb | inactive | 0.1 | 69 | 400 Kb | 1 / 5014 bp | 7.24 | 19.94 | 5.14 |
| 1SNP/2Kb | inactive | 0.1 | 100 | 200 Kb | 1 / 2106 bp | 8.57 | 22.77 | 5.53 |
| 1SNP/Kb | inactive | 0.1 | 100 | 200 Kb | 1/ 1233 bp | 7.58 | 23.92 | 5.83 |
| all SNPs | inactive | 0.1 | 100 | 200 Kb | 1 / 512 bp | 12.72 | 24.00 | 7.28 |
| TAG pairwise | inactive | 0.1 | 1000 | 200 Kb | 1 / 1545 bp | 7.2 | 17.83 | 5.54 |
| TAG pairwise | active | 0.1 | 100 | 200 Kb | 1/1980 | 5.65 | 18.67 | 5.52 |
| 1SNP/2Kb | inactive | 0.01 | 100 | 200 Kb | 1/2000 bp | 16.01 | 21.04 | 6.69 |
| 1SNP/Kb | inactive | 0.01 | 100 | 200 Kb | 1/1013 bp | 20.56 | 21.76 | 7.51 |
