# Supplementary material for: A New Method to Reconstruct Recombination Events at a Genomic Scale
Source: PLoS Comput Biol. 2010 Nov 24;6(11):e1001010. doi: 10.1371/journal.pcbi.1001010 (PMC2991245; doi:10.1371/journal.pcbi.1001010)
Supplement: Table S2 — Percentage values on the number of times each of the simulated event is either not detected, detected as 1 recombination or as 2 recombinations. The percentage values are calculated over 1,000 in silico simulations. (0.03 MB DOC) [file pcbi.1001010.s008.doc]

|  | % not detected as recombination | | % detected as 1 recombination | | % detected as 2 recombination | |
| --- | --- | --- | --- | --- | --- | --- |
| mergepats parameter | active | inactive | active | inactive | active | inactive |
| gene conversion (1 SNP) | 99.2 | 97.7 | 0.8 | 2.3 | 0 | 0 |
| gene conversion (3 SNPs) | 96.2 | 92.6 | 3.7 | 7.3 | 0.1 | 0.1 |
| gene conversion (5 SNPs) | 90 | 88 | 9.8 | 12 | 0.2 | 0 |
| gene conversion (10 SNPs) | 76.2 | 74 | 23.7 | 25.6 | 0.1 | 0.4 |
| recurrent mutation | 89 | 78.5 | 10.8 | 21.4 | 0.2 | 0.1 |
| phasing errors | 57.3 | 50.9 | 12.1 | 14.6 | 30.4 | 34.5 |
