# Supplementary material for: A New Method to Reconstruct Recombination Events at a Genomic Scale
Source: PLoS Comput Biol. 2010 Nov 24;6(11):e1001010. doi: 10.1371/journal.pcbi.1001010 (PMC2991245; doi:10.1371/journal.pcbi.1001010)
Supplement: Table S3 — Number of recombinations detected in each of the 18 regions in the male dataset, female dataset and female dataset when removing putative phasing errors. Females were phased using both PHASE and fastPHASE without using male phase information. (0.05 MB DOC) [file pcbi.1001010.s009.doc]

| REGIONS | MALE | FEMALE | FEMALE | MALE cleaned | FEMALE cleaned | FEMALE cleaned |
| --- | --- | --- | --- | --- | --- | --- |
| phasing method |  | PHASE | fastPHASE |  | PHASE | fastPHASE |
| reg 1 | 442 | 432 | 473 | 364 | 376 | 359 |
| reg 2 | 237 | 246 | 290 | 221 | 234 | 248 |
| reg 3 | 58 | 77 | 77 | 58 | 75 | 73 |
| reg 4 | 57 | 59 | 64 | 55 | 59 | 62 |
| reg 5 | 269 | 269 | 319 | 257 | 255 | 293 |
| reg 6 | 24 | 31 | 28 | 24 | 31 | 26 |
| reg 7 | 149 | 166 | 178 | 139 | 166 | 162 |
| reg 8 | 224 | 204 | 232 | 216 | 198 | 228 |
| reg 9 | 298 | 312 | 353 | 284 | 300 | 315 |
| reg 10 | 99 | 110 | 117 | 97 | 110 | 103 |
| reg 11 | 126 | 111 | 133 | 114 | 107 | 123 |
| reg 12 | 293 | 285 | 321 | 283 | 279 | 293 |
| reg 13 | 75 | 77 | 78 | 73 | 73 | 76 |
| reg 14 | 44 | 38 | 44 | 44 | 38 | 42 |
| reg 15 | 370 | 324 | 388 | 326 | 308 | 326 |
| reg 16 | 262 | 256 | 287 | 236 | 242 | 243 |
| reg 17 | 252 | 264 | 293 | 228 | 240 | 257 |
| reg 18 | 319 | 322 | 399 | 305 | 306 | 351 |
| ALL | 3598 | 3583 | 4074 | 3324 | 3397 | 3580 |
