# Supplementary material for: A New Method to Reconstruct Recombination Events at a Genomic Scale
Source: PLoS Comput Biol. 2010 Nov 24;6(11):e1001010. doi: 10.1371/journal.pcbi.1001010 (PMC2991245; doi:10.1371/journal.pcbi.1001010)
Supplement: Table S4 — The main characteristics of 18 X-chromosome regions. From left to right: start position and end position in base pairs (based on NCBI Build 36 assembly), length of each in base pairs, number of SNPs (N SNPs), number of haplotypes (N haplo), recombination rate calculated by means of Ldhat, Number of recombinations detected, number of recotypes, average number of recombinations detected by IRiS per Kb. (0.06 MB DOC) [file pcbi.1001010.s010.doc]

| region | start (bp) | end (bp) | Length (bp) | N snps | N haplo | Rec Rate (4Ne/bp) | N rec | N reco | n_rec/Kb |
| --- | --- | --- | --- | --- | --- | --- | --- | --- | --- |
| reg1 | 22505979 | 22728622 | 222643 | 95 | 485 | 1.34 | 442 | 367 | 1.99 |
| reg2 | 23071760 | 23213016 | 141256 | 97 | 375 | 1.06 | 237 | 208 | 1.68 |
| reg3 | 25715611 | 26016381 | 300770 | 83 | 208 | 0.27 | 58 | 59 | 0.19 |
| reg4 | 35038017 | 35504132 | 466115 | 84 | 170 | 0.23 | 57 | 57 | 0.12 |
| reg5 | 38875482 | 39480082 | 604607 | 179 | 473 | 0.44 | 269 | 211 | 0.44 |
| reg6 | 84704863 | 84952842 | 247979 | 80 | 81 | 0.11 | 24 | 24 | 0.10 |
| reg7 | 86338463 | 86609425 | 270962 | 91 | 372 | 0.65 | 149 | 146 | 0.55 |
| reg8 | 87288915 | 87838907 | 549992 | 205 | 453 | 0.54 | 224 | 187 | 0.41 |
| reg9 | 93522874 | 94555707 | 1032833 | 183 | 478 | 0.37 | 298 | 223 | 0.29 |
| reg10 | 112181012 | 112602418 | 421406 | 92 | 241 | 0.24 | 99 | 98 | 0.23 |
| reg11 | 116631417 | 116865805 | 234388 | 82 | 324 | 0.53 | 126 | 123 | 0.54 |
| reg12 | 120875730 | 121450338 | 574608 | 157 | 401 | 0.46 | 293 | 237 | 0.51 |
| reg13 | 125833172 | 126301999 | 468827 | 91 | 169 | 0.19 | 75 | 74 | 0.16 |
| reg14 | 126499106 | 126892013 | 392907 | 84 | 84 | 0.09 | 44 | 44 | 0.11 |
| reg15 | 140883556 | 141050268 | 166712 | 99 | 494 | 1.68 | 370 | 327 | 2.22 |
| reg16 | 141376625 | 141647366 | 270741 | 89 | 462 | 0.95 | 262 | 226 | 0.97 |
| reg17 | 143563468 | 143896320 | 332852 | 97 | 414 | 0.61 | 252 | 235 | 0.76 |
| reg18 | 144769060 | 145266667 | 497607 | 164 | 480 | 0.64 | 319 | 248 | 0.64 |
| ALL |  |  | 7197205 | 2052 |  |  | 3598 |  |  |
