# Supplementary material for: A New Method to Reconstruct Recombination Events at a Genomic Scale
Source: PLoS Comput Biol. 2010 Nov 24;6(11):e1001010. doi: 10.1371/journal.pcbi.1001010 (PMC2991245; doi:10.1371/journal.pcbi.1001010)
Supplement: Supplementary file 8 [file pcbi.1001010.s003.doc]

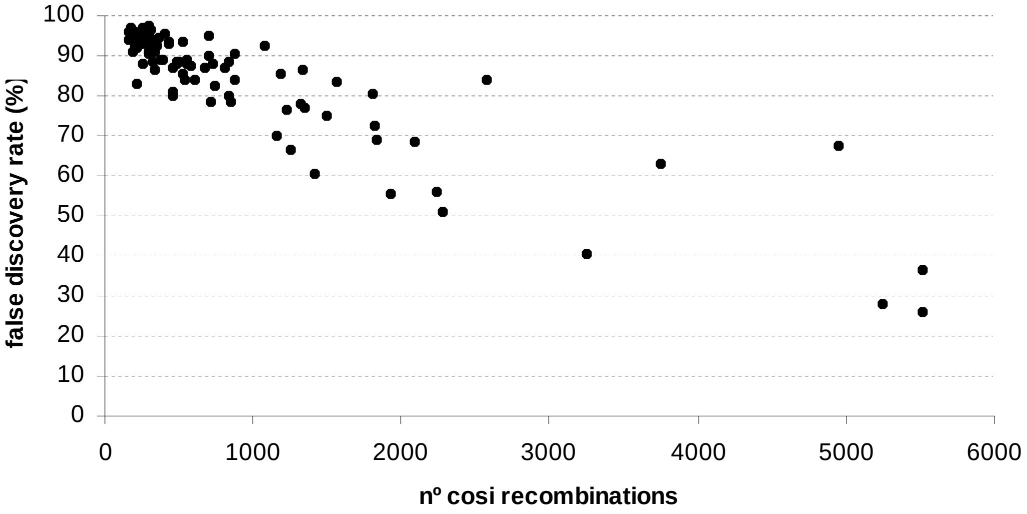
false discovery rate (%)
100
90
80
70
60
50
40
30
20
10
0
0
1000
2000
3000
4000
5000
6000
nº cosi recombinations
