# Supplementary material for: A New Method to Reconstruct Recombination Events at a Genomic Scale
Source: PLoS Comput Biol. 2010 Nov 24;6(11):e1001010. doi: 10.1371/journal.pcbi.1001010 (PMC2991245; doi:10.1371/journal.pcbi.1001010)
Supplement: Supplementary file 9 [file pcbi.1001010.s004.doc]

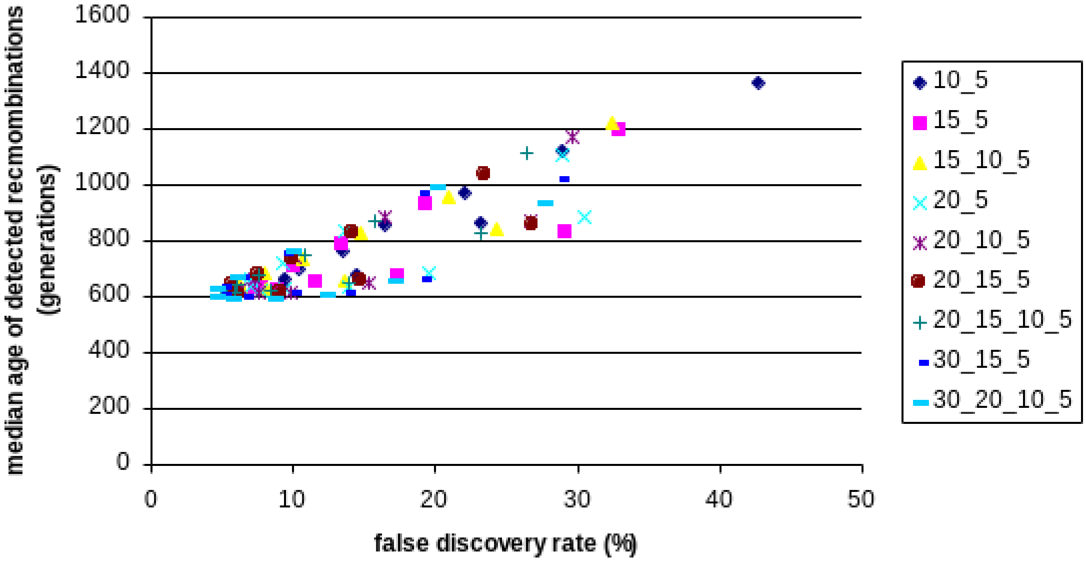

median age of detected recmombinations (generations)
1600
1400
1200
1000
800
600
400
200
0
0
10
20
30
40
50
false discovery rate (%)
10_5
15_5
15_10_5
20_5
20_10_5
20_15_5
20_15_10_5
30_15_5
30_20_10_5
