# Supplementary material for: A New Method to Reconstruct Recombination Events at a Genomic Scale
Source: PLoS Comput Biol. 2010 Nov 24;6(11):e1001010. doi: 10.1371/journal.pcbi.1001010 (PMC2991245; doi:10.1371/journal.pcbi.1001010)
Supplement: Supplementary file 10 [file pcbi.1001010.s005.doc]

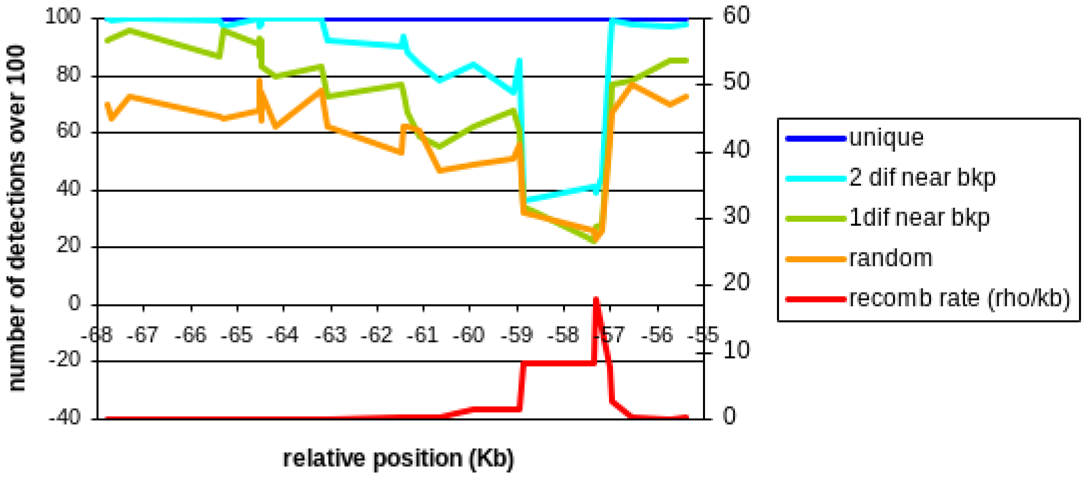

number of detections over 100
100
80
60
40
20
0
-20
-40
60
50
40
30
20
10
0
-68 -67 -66 -65 -64 -63 -62 -61 -60 -59 -58 -57 -56 -55
relative position (Kb)
unique
2 dif near bkp
1dif near bkp
random
recomb rate (rho/kb)
